# Therapeutic effects of Reiki on interventions for anxiety: a meta-analysis

**DOI:** 10.1186/s12904-024-01439-x

**Published:** 2024-06-13

**Authors:** Xiulan Guo, Yue Long, Zhikai Qin, Yongtao Fan

**Affiliations:** Capital University of Physical Education and Sports, Beijing, 100191 China

**Keywords:** Reiki therapy, Anxiety, Quality of life, Meta-analysis

## Abstract

**Purpose:**

This study aimed to assess the therapeutic efficacy of Reiki therapy in alleviating anxiety.

**Methods:**

In adherence to academic standards, a thorough search was conducted across esteemed databases such as PubMed, Web of Science, Science Direct, and the Cochrane Library. The primary objective of this search was to pinpoint peer-reviewed articles published in English that satisfied specific criteria: (1) employing an experimental or quasi-experimental study design, (2) incorporating Reiki therapy as the independent variable, (3) encompassing diverse patient populations along with healthy individuals, and (4) assessing anxiety as the measured outcome.

**Results:**

The study involved 824 participants, all of whom were aged 18 years or older. Reiki therapy was found to have a significant effect on anxiety intervention(SMD=-0.82, 95CI -1.29∼-0.36, *P* = 0.001). Subgroup analysis indicated that the types of subjects (chronically ill individuals and the general adult population) and the dosage/frequency of the intervention (≤ 3 sessions and 6–8 sessions) were significant factors influencing the variability in anxiety reduction.

**Conclusion:**

Short-term Reiki therapy interventions of ≤ 3 sessions and 6–8 sessions have demonstrated effectiveness in reducing health and procedural anxiety in patients with chronic conditions such as gastrointestinal endoscopy inflammation, fibromyalgia, and depression, as well as in the general population. It is important to note that the efficacy of Reiki therapy in decreasing preoperative anxiety and death-related anxiety in preoperative patients and cancer patients is somewhat less consistent. These discrepancies may be attributed to individual pathophysiological states, psychological conditions, and treatment expectations.

**Supplementary Information:**

The online version contains supplementary material available at 10.1186/s12904-024-01439-x.

## Introduction

Individuals undergoing hospital examinations may experience preoperative anxiety, stress, and fear due to concerns about the examination procedure, diagnostic results, discomfort during the procedure (such as vomiting, nausea, swelling, and pain), and potential associated risks [1, 2]. Procedural anxiety, defined as the anxious and uneasy emotions experienced when encountering specific procedures or processes, can stem from uncertainty, fear, or concern about upcoming medical procedures [3]. Preoperative anxiety is a common manifestation of procedural anxiety, leading to heightened stress and anxiety in patients before medical examinations and potentially impacting the examination process, posing challenges for healthcare providers [4, 5]. Individuals with chronic pain are more prone to developing psychiatric comorbidities compared to the general population [6], with chronic pain closely linked to depression [7] and symptoms of health anxiety [8]. While death is an inevitable part of life, its uncertain nature can evoke feelings of anxiety [9, 10]. Patients facing incurable illnesses directly face mortality [11], and those diagnosed with late-stage diseases like cancer may experience heightened anxiety related to death [12]. The field of psychiatry encompasses various types of anxiety disorders, presenting multiple avenues for complementary therapies to aid in anxiety management.

Reiki, a contemporary spiritual energy therapy reintroduced by Mikao Usui in late 19th-century Japan, involves channeling healing energy through the body’s chakras [13]. Chakras, the energy centers that regulate the body’s energy, are situated along the spine and support energy circulation throughout the body [14]. In Reiki, the focused transmission of healing energy aims to address imbalances in energy fields linked to physical, emotional, or psychological sources of distress [15, 16]. Reiki is viewed as a technique to harmonize the body, mind, and spirit by stimulating the parasympathetic nervous system [17, 18]. Reiki, an ancient Japanese practice, can be applied at home or remotely, utilizing intentional guided healing energy [19]. This natural therapy involves transmitting energy to the basic seven chakras, including the head, neck, chest, abdomen, and groin. The energy is transmitted through direct touch or non-contact, with each chakra receiving 3 to 5 min of attention [20]. Despite its historical roots, Reiki has gained popularity among more than 1.5 million Americans and continues to attract new practitioners [21]. In contemporary society, there is a growing emphasis on improving quality of life, reducing fatigue, and managing anxiety. These aspects play a crucial role in overall well-being, encompassing physical health, mental well-being, and the broader impact of social and environmental factors. Many individuals experience increasing levels of fatigue and stress due to work demands, family pressures, and societal expectations as they strive for personal and professional success. These challenges can have detrimental effects on both physical and mental health, contributing to conditions like anxiety and depression. Reiki, as an energy-based touch therapy, offers a means to rebalance and revitalize the body’s energy system when it is disrupted by stress or negative emotions [22].

Reiki, a low-risk and cost-free method, has shown to improve anxiety and stress reduction, as well as enhance overall quality of life [23, 24]. Some studies suggest that integrating Reiki therapy into holistic care can address the interconnectedness of body, mind, and soul, contributing to overall well-being. It could also be a valuable independent function provided by nurses [25, 26]. The National Center for Complementary and Alternative Medicine categorizes Reiki as a biofield therapy and energy therapy [27]. Traditional medical treatments, such as drug therapy, can be costly and come with potential side effects that may hinder both physical pain relief and psychological anxiety alleviation [28]. As a result, many patients can benefit from alternative and complementary therapeutic approaches alongside traditional surgical and drug treatments. Reiki, which translates to universal life energy, is an affordable, side-effect-free, and easily applicable complementary and integrative therapy. Previous meta-analyses have investigated the effects of Reiki on pain in both cancer patients and the general population, demonstrating a decrease in patient suffering [28, 29]. Expanding on this research, the objective of this study is to conduct a systematic review on the impact of Reiki on patient anxiety.

## Materials and methods

### Study design

This article presents a systematic review of randomized controlled trials (RCTs) that were conducted in accordance with the Preferred Reporting Items for Systematic Reviews and Meta-Analyses (PRISMA) guidelines [30]. The protocol for this review was registered in the International Prospective Register of Systematic Reviews (PROSPERO) before screening search results (Registration Number: CRD42023483969), and the reporting of the study adheres to the PRISMA statement.

### Study inclusion criteria

This study includes controlled trials (RCTs) of Reiki therapy with a minimum of one session. The Reiki therapy should be compared with a placebo group or blank control. The participants in the selected studies should be adults (aged 18 years or older) without any restrictions based on gender, race, or socioeconomic background. The study can involve various types of patients such as gastrointestinal endoscopy patients, cancer patients, surgery patients, chronic disease patients, and depression patients, as well as normal adults. The anxiety levels will be assessed using anxiety scales in the eligible studies. Only studies in English and in full-text format will be considered. Descriptive reviews, preclinical studies, duplicate studies, editorials or opinion articles, grey literature, and conference papers will not be included. Systematic reviews and study protocols that do not meet the inclusion criteria will be evaluated as guidelines and cited when appropriate.

### Search strategy

This meta-analysis aimed to assess the effectiveness of Reiki therapy in reducing anxiety. Searches were conducted in the PubMed, PsycINFO, and Cochrane Library databases from January 1, 2005, to November 11, 2023, to ensure a comprehensive selection of relevant studies. The search terms included ‘Reiki therapy’ OR ‘Reiki intervention’ AND ‘Anxiety’ AND (‘Controlled Trial’ OR ‘Randomized Controlled Trial’ OR ‘Clinical Trial’ OR ‘Controlled Study’ OR ‘Comparative Study’ OR ‘Placebo-Controlled Trial’) AND (‘Procedural Anxiety’ OR ‘Health Anxiety’ OR ‘Death Anxiety’).

### Study selection process

Search results were imported into Zotero 6.0. After removing duplicates, two reviewers independently screened the titles and abstracts of the studies. Studies that did not meet the eligibility criteria were excluded. Full texts of all relevant studies were obtained, downloaded, and further assessed for eligibility. Any disagreements between the two reviewers regarding the inclusion of specific studies were resolved through consultation with a third independent reviewer to minimize bias in the decision of whether to include certain studies. Data extraction was independently performed by two reviewers. Discrepancies were resolved by consulting the aforementioned third independent reviewer.

### Data extraction

The data extracted from the selected studies covered various areas including authorship, publication year, sample size, participant age and gender, study design, intervention description (including method, frequency and duration, and key components), control group, outcome measures and time points, results, dropout rates, and handling of missing data.

### Effect size measurement

The inclusion criteria for study outcomes involved evaluating the average difference between the Reiki therapy intervention group and the control group at the assessment endpoint. Data were extracted and recorded independently by two authors, with any discrepancies resolved through consensus or consultation with a third reviewer. Manuscripts were included in the meta-analysis only if the results of the anxiety scale were adequately reported.

### Data synthesis

Using the Der Simonian-Laird random-effects and fixed-effects models (depending on heterogeneity) and STATA software (version 15), we calculated the summary values of the weighted mean differences between the Reiki treatment group and the control group. We also calculated the corresponding 95% confidence intervals (CIs). The effect size estimation was weighted by the inverse of its variance, and we used Hedges’ g statistic to calculate the effect size of the standardized mean difference and its corresponding 95% CI. Hedges’ g size interpretations were as follows: small (g = 0.3), medium (g = 0.5), and large (g = 0.8). To assess heterogeneity, we used the chi-square test to evaluate the null hypothesis that all studies assessed the same effect. We quantified the total variation consistent with heterogeneity among studies using the inconsistency index (i.e., I^2^), which ranges from 0 to 100%. We considered a *p*-value < 0.10 from the chi-square test and I^2^ > 50% as signs of significant heterogeneity [31]. We generated a funnel plot, using the effect size for each trial against standard error, to assess potential publication bias. The asymmetry of the funnel plot was evaluated through Egger’s small-sample effect test based on regression.

### Risk of bias (quality) assessment

The quality of each included study was assessed using the Cochrane Risk of Bias tool, which evaluated random sequence generation, allocation concealment, blinding of participants and personnel, blinding of outcome assessment, incomplete outcome data, selective reporting, and other biases [32]. Two authors independently conducted the quality assessments, and any discrepancies were resolved by a third reviewer.

## Results

### Study selection

**Fig. 1:**
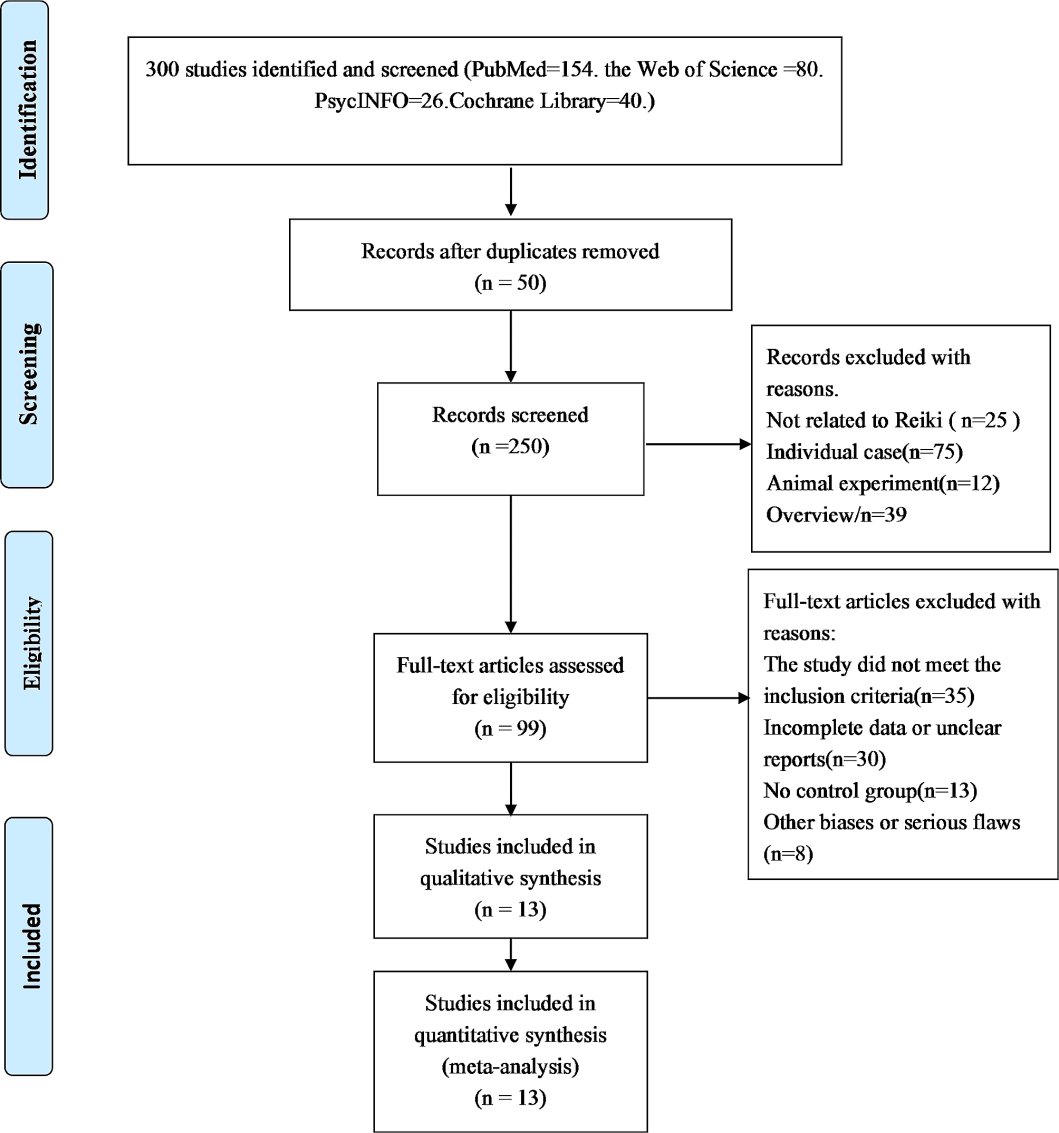
Flowchart of the study

A total of 300 articles were initially identified from four databases, with 50 duplicates. After screening the titles and abstracts of 250 articles, 151 were excluded as they did not meet the inclusion criteria. The remaining 99 articles that met the criteria underwent a full-text review. Out of these, 86 articles were excluded, and the reasons for their exclusion are detailed in Fig. 1. Ultimately, this systematic review included 13 studies [13, 23, 33–43].

### Risk of bias of included studies

**Fig. 2:**
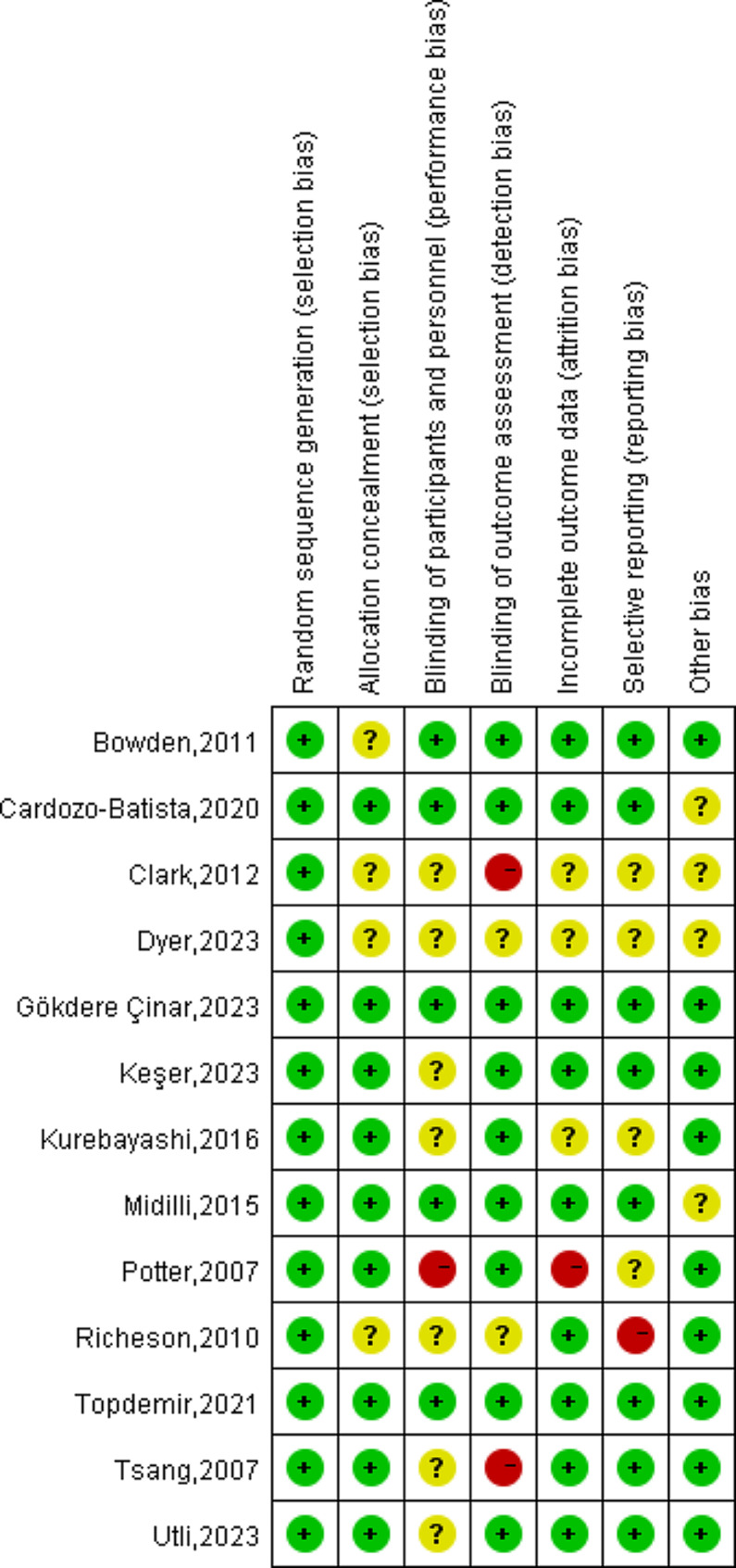
Risk of bias summary

**Fig. 3:**
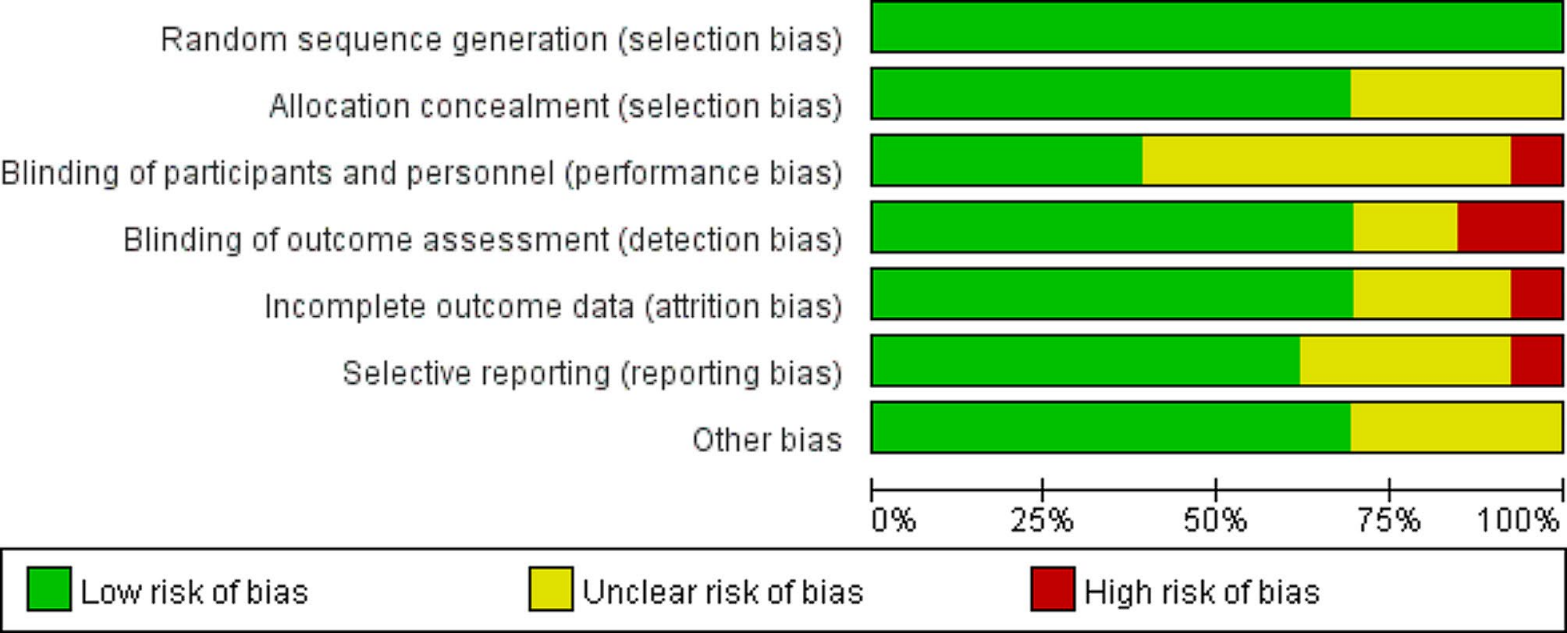
Risk of bias graph

All studies included in this review adequately described the generation of random allocation sequences, indicating a low risk of selection bias associated with sequence generation. Nine trials provided specific details on maintaining the confidentiality of sample allocation, categorizing them as low risk [13, 23, 33, 35–39, 41]. However, the remaining studies lacked detailed descriptions of procedures and clear information, leading to an unclear classification. Regarding performance bias, the nature of these trials made blinding of participants or Reiki practitioners challenging [23, 33–35, 37, 38, 40, 43]. However, it is crucial to blind the outcome assessment in Reiki intervention studies. Nevertheless, four studies reported measures taken for blinding outcome assessment, while for the remaining, it was unclear or not specified [23, 34, 40, 43]. The assessment of attrition and reporting bias was heavily influenced by these two studies [40, 43]. A summary and a graph of the risk of bias are provided in Figs. 2 and 3, respectively.

### Study characteristics

Table 1 summarizes the key characteristics of 13 controlled experimental studies involving a total of 824 participants, which investigate the effectiveness of Reiki therapy for treating anxiety. Notably, a larger number of studies have been conducted in the United States [34, 35, 40, 43] and Turkey [13, 33, 36, 38, 39] on the application of Reiki therapy. This paper assesses the selected studies based on their research goals, study features, outcome measures, and main findings.

**Table 1:** Characteristics of the studies in the systematic review and meta-analysis

Author/Year	Author’s country	Research design	Sample size (T/C)	Age range	Subject type	Intervention design (T/C)	Exercise prescription	Evaluation tools/content
Utli,2023 [33]	Turkey	RCT	53/53	49.6 ± 10.2	Chronically ill	Reiki/Control group	25 min/times, 1 / week, 1 week	SAI
Tsang,2007 [23]	Canada	RCT	8/8	59 ± 15.23	Cancer patient	Reiki/Control group	45 min/times, 5 / week, 1 week	Esas
Topdemir,2021 [13]	Turkey	RCT	105/105	36.67 ± 13.62	Surgical patient	Reiki/Control group	60 min/times, 2 / week, 1 week	STAI
Richeson,2010 [34]	America	RCT	12/8	Community elderly	Community elderly	Reiki/Control group	45 min/times, 1 / week, 8 weeks	HAM-A
Potter,2007 [35]	America	RCT	17/15	Middle age women	Middle age women	Reiki/Control group	50 min/times, 2 / week, 1 week	STAI
Midilli,2015 [36]	Turkey	RCT	45/45	18–45	Surgical patient	Reiki/Control group	30 min/times, 2 / week, 1 week	SAI
Kurebayashi,2016 [37]	Brazil	RCT	38/33	18–45	Normal adult	Reiki + massage/Control group	45 min/times, 2 / week, 4 weeks	IDATE
Keşer,2023 [38]	Turkey	RCT	34/33	37.47 ± 10.45	Chronically ill	Reiki/Control group	20 min/times, 2 / week, 1 week	SAS
Gökdere Çinar,2023 [39]	Turkey	RCT	25/25	43.56 ± 9.52	Chronically ill	Reiki/Control group	30 min/times, 1 / week, 4 weeks	STAI
Dyer,2023[41]	America	RCT	40/39	35.56 ± 9.32	Normal adult	Reiki/Control group	20 min/times, 4 / week, 1 week	MYMOP
Cardozo-Batista,2020 [41]	Brazil	RCT	10/11	Medical staff	Chronically ill	Reiki/Control group	65 min/times, 1 / week, 8 weeks	DASS-21
Bowden,2011 [42]	Britain	RCT	20/20	39.4 ± 10.36	Normal adult	Reiki/Control group	30 min/times, 6 / week, 4 weeks	HADS
Clark,2012 [43]	America	RCT	7/5	59.04 ± 8.56	Cancer patient	Reiki/Meditation	60 min/times, 1 / week, 6 weeks	QLN

Notes: Intervention Group (T); Control Group (C); Randomized controlled trial (RCT); State Anxiety Inventory (SAI); Hamilton Anxiety Scale (HAM-A); State-Trait Anxiety Inventory (STAI); Hamilton Anxiety Scale (HAM-A); State Anxiety Scale (SAI); Trace State Anxiety Inventory (IDATE); State Anxiety Subscale (SAS); Measure Yourself Medical Outcome Profile (MYMOP); Depression, Anxiety and Stress Scale-21 (DASS-21); Hospital Anxiety and Depression Scale (HADS); Quality of life and neurotoxicity(QLN);

### Meta-analysis

**Fig. 4:**
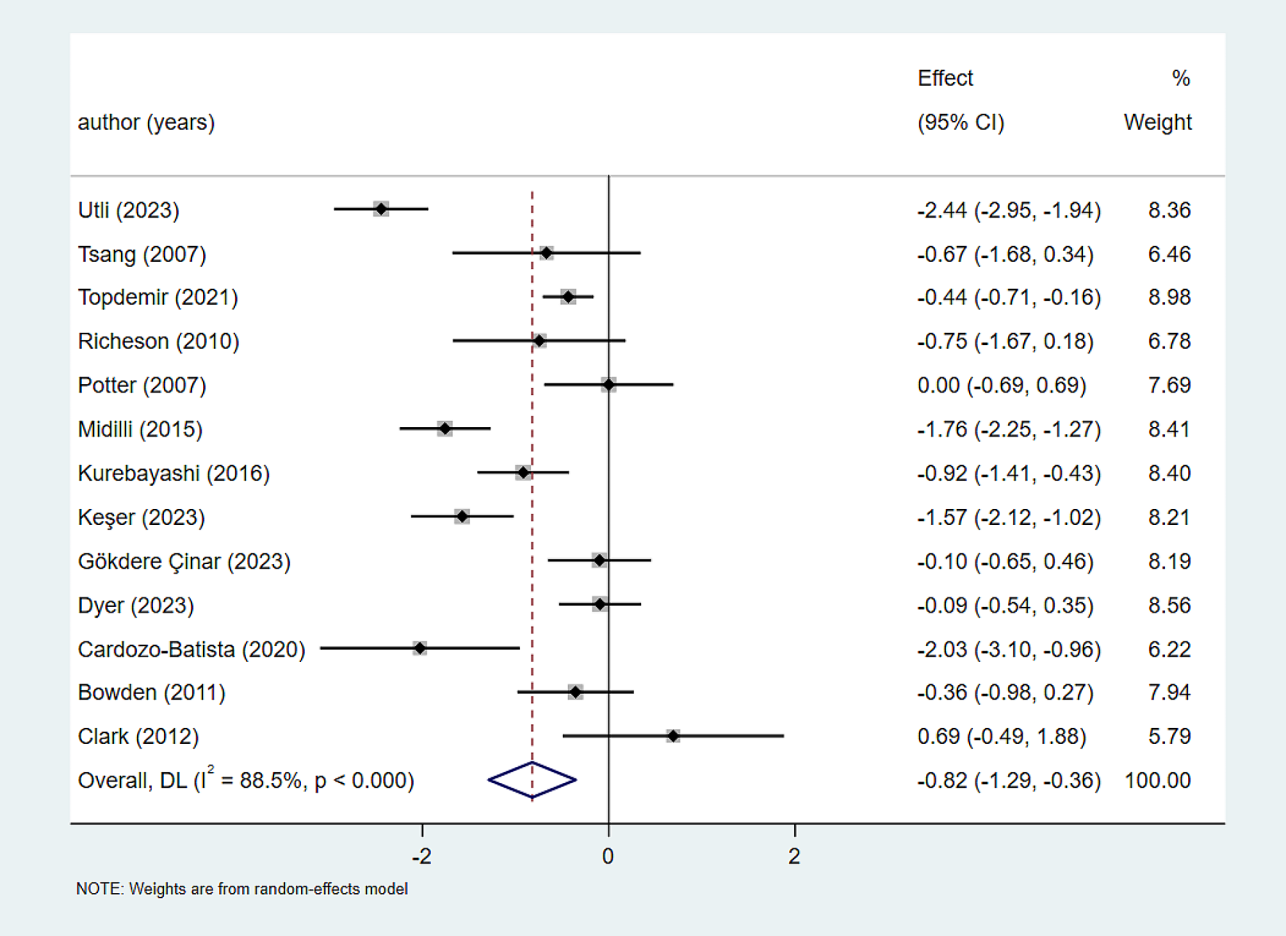
Forest plot of Reiki therapy in the intervention of anxiety

**Fig. 5:**
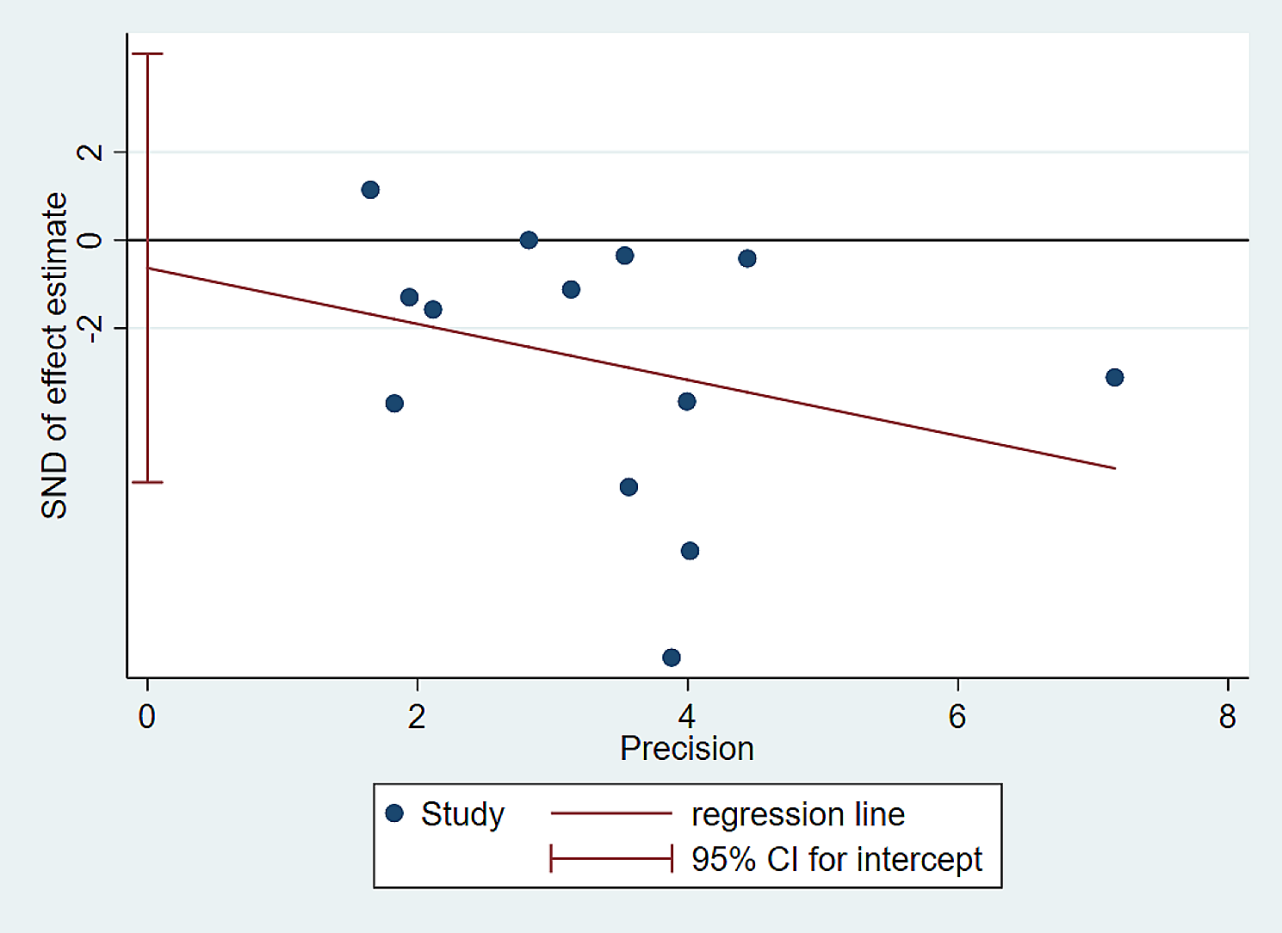
Publication bias plot of Reiki therapy in the intervention of anxiety

In this systematic evaluation, we assessed a total of 13 studies [13, 23, 33–43] involving 824 patients to evaluate the effects of Reiki therapy on anxiety relief. We observed considerable heterogeneity among the studies (I^2^ = 88.5%, *P* < 0.000), which led us to use a random-effects model for analysis. The results showed a significant impact of Reiki therapy on anxiety intervention(SMD=-0.82, 95CI -1.29∼-0.36, *P* = 0.001). Refer to Fig. 4 for a graphical representation. As I^2^ > 50% (I^2^ = 88.5%, *P* < 0.000), we performed an Egger regression to detect publication bias. Egger regression is a statistical method used to evaluate if study outcomes are influenced by the small sample effect [44]. The results of the Egger test (t=-0.29, *P* = 0.779) indicated no evidence of publication bias. Please refer to Fig. 5 for more details.

### Subgroup analysis

To investigate the factors influencing the effectiveness of Reiki therapy in alleviating anxiety, subgroup analyses were conducted based on different patient types, intervention durations, and intervention frequencies.

Subgroup analysis results on anxiety indicate that factors such as the type of subjects (chronically ill individuals and the general adult population) and the dosage/frequency of intervention (≤ 3 and 6–8 sessions) significantly contribute to the heterogeneity affecting anxiety relief. Please refer to Table 2 for more details. Short-term interventions (≤ 3 sessions) and moderate-frequency Reiki therapy (6–8 sessions) have shown effectiveness in alleviating health and procedural anxiety in patients with chronic conditions (e.g., those undergoing gastrointestinal endoscopy, fibromyalgia, and depression) and the general adult population. However, it is important to note that the effectiveness of Reiki therapy in reducing preoperative and death anxiety in preoperative and cancer patients is relatively lower.

**Table 2:** Subgroup analysis results of Reiki therapy in the treatment of anxiety

Dimensionality	sort	Number of studies/papers	I^2^	Effect model	SMD and 95%CI	*P*
Subject type	Chronically ill	4	95%	Random	-1.75(-3.20,-0.29)	0.018
	Surgical patient	2	95.3%	Random	-1.08(-2.38,0.21)	0.102
	Cancer patient	2	65.8%	Random	-0.03(-1.36,1.31)	0.970
	Normal adult population	4	49.7%	Fix	-0.41(-0.79,-0.03)	0.035
Intervention time/minute	≤ 30	6	93.3%	Random	-1.06(-1.88,-0.23)	0.012
	45–60	7	64.4%	Random	-0.58(-1.02,-0.15)	0.009
Intervention dose/frequency	≤ 3	5	94.2%	Random	-1.25(-2.12,-0.38)	0.005
	4–6	5	0.0%	Fix	-0.15(-0.43,0.13)	0.289
	6–8	3	48.6%	Fix	-1.13(-1.77,-0.49)	0.001

## Discussion

This systematic review includes 13 studies [13, 23, 33–43], which evaluate the effectiveness of Reiki therapy in reducing anxiety among 824 patients. The results show a significant impact of Reiki intervention on anxiety(SMD=-0.82, 95CI -1.29∼-0.36, *P* = 0.001). Overall, short-term interventions (≤ 3 sessions) and moderate-frequency Reiki treatments (6–8 sessions) have shown effectiveness in reducing health and procedural anxiety in patients with chronic conditions (such as those undergoing gastrointestinal endoscopy, fibromyalgia, and depression) as well as in the general adult population. Previous meta-analyses have investigated the effects of Reiki on pain relief. Avci et al. [28] conducted a meta-analysis of 7 relevant studies, concluding that the use of Reiki can decrease pain levels in cancer patients. In a similar vein, Thrane et al. [45] examined 20 studies and found evidence that Reiki can help alleviate pain. Anxiety, as defined by the American Psychological Association, is marked by physiological reactions such as fear, anxious thoughts, and increased blood pressure [46]. In the context of mitigating anxiety and stress levels, Reiki, a complementary energy therapy, has been found to enhance pharmacological treatments [36]. Various reviews, such as those conducted by Billot et al., have demonstrated the efficacy of Reiki in reducing anxiety across diverse groups. These groups encompass a range of individuals, from those in good health to individuals experiencing chronic pain, post-hysterectomy patients, women undergoing breast biopsies, stage I to IV cancer patients, and older adults living in the community. Nonetheless, a single study indicates that Reiki may not have a significant impact on anxiety levels in prostate cancer patients undergoing radiation therapy [18, 47–51]. Gastroscopy, an invasive procedure that can induce anxiety in some individuals due to the discomfort of a foreign object in their body, often deters people from undergoing upper gastrointestinal endoscopy, resulting in missed examinations [52]. Reiki has been shown as an adjunctive sedative treatment to help alleviate pre-gastroscopy anxiety, reducing the need for sedatives, lowering the risk of complications, and enhancing patient safety [33]. Fibromyalgia, a chronic syndrome characterized by widespread musculoskeletal pain and tender points [53], was treated with Reiki therapy by Gökdere Çinar et al. The study reported positive effects including pain relief, improved quality of life, and reduced trait anxiety levels [39]. Richeson et al. [34] investigated the efficacy of Reiki as a complementary treatment for pain, depression, and anxiety in community-dwelling elderly individuals. Their findings revealed significant improvements in symptom relief, suggesting that Reiki may be a beneficial intervention for this population. Additionally, previous studies have shown that Reiki can reduce anxiety and depression in college students [42] and adolescents [54]. Overall, the use of Reiki as an adjunct therapy has shown promise in alleviating pain and anxiety in diverse populations.

Clark et al. [43] discovered that Reiki had positive impacts on psychological distress and quality of life in patients with chemotherapy-induced peripheral neuropathy. Tsang et al. [23] explored the potential benefits of Reiki in reducing cancer-related fatigue (CRF) and enhancing overall well-being in cancer patients. Their results indicated that Reiki had a moderate effect on reducing CRF and significantly improving fatigue levels, daily pain, anxiety, and overall quality of life. Topdemir et al. have conducted a study with preoperative patients, where the experimental group showed no change in state anxiety scores, while the control group experienced an increase in state anxiety scores [13]. A small-scale pilot study with blinding and placebo control demonstrated that Reiki could reduce anxiety levels in hospitalized patients undergoing surgical treatment [55]. Additionally, Cassidy et al. [56] conducted a study and found that combining Reiki with music significantly decreased preoperative anxiety in patients when compared to the use of music alone. The subgroup analysis conducted in this study revealed that Reiki therapy did not show a statistically significant reduction in anxiety among cancer patients and preoperative patients. This variance could be linked to individual pathological and physiological conditions, psychological states, and treatment expectations. It is advisable to conduct additional research specifically targeting Reiki therapy in cancer and preoperative patients to delve into its effectiveness within these particular groups and examine possible influencing factors, including patients’ beliefs, attitudes, and the severity of their conditions. The duration of the intervention also plays a role in anxiety relief. Olsen et al. found that the Reiki group had a moderate effect on pain reduction on the first day [24]. Thrane et al. [45] suggested that interventions lasting 26 weeks or longer could highlight differences between the Reiki group and the sham Reiki group. These results align with the subgroup analysis in this study, showing that short-term interventions (≤ 3 times) and 6–8 sessions of Reiki therapy yield positive outcomes for anxiety relief.

### Limitations

This systematic evaluation of Reiki therapy for anxiety relief acknowledges certain inherent limitations. The limited number of studies prevented a meta-analysis for a specific population, prompting the exploration of factors influencing treatment effects through subgroup analysis. Notably, fewer controlled studies were available for cancer patients in the subgroup analysis, potentially impacting the results. The parameters used for grouping in the subgroup analysis had limitations in identifying factors influencing intervention effects. Additionally, this review only considered English-language controlled experiments, possibly excluding pertinent literature published in other languages.

## Conclusions

From an integrative perspective, research has shown that short-term interventions (≤ 3 sessions) and moderate-frequency Reiki therapy (6–8 sessions) can effectively reduce health and procedural anxiety in patients with chronic conditions like gastrointestinal endoscopy, fibromyalgia, and depression, as well as in the general adult population. It is important to note that the effectiveness of Reiki therapy in reducing preoperative and death anxiety in preoperative patients and cancer patients appears to be lower. Further studies focusing on Reiki therapy in cancer and preoperative patients are recommended to better understand its effects in these specific populations and explore potential influencing factors, such as patients’ beliefs, attitudes, and disease severity.

### Electronic supplementary material

Below is the link to the electronic supplementary material.

Supplementary Material 1

Supplementary Material 2

## Data Availability

All data and materials can be accessed by contacting the first author.
