# Supplementary material for: Therapeutic effects of Reiki on interventions for anxiety: a meta-analysis
Source: BMC Palliat Care. 2024 Jun 13;23:147. doi: 10.1186/s12904-024-01439-x (PMC11170819; doi:10.1186/s12904-024-01439-x)
Supplement: Supplementary file 2 — Supplementary Material 2 [file 12904_2024_1439_MOESM2_ESM.docx]

| **Section and Topic** | **Item #** | **Checklist item** | **Location where item is reported** |
| --- | --- | --- | --- |
| **TITLE** | | |  |
| Title | 1 | Identify the report as a systematic review. | The report was identified as a systematic review titled "Therapeutic Effects of Reiki on Interventions for Anxiety: A Meta-Analysis." |
| **ABSTRACT** | | |  |
| Abstract | 2 | See the PRISMA 2020 for Abstracts checklist. | This abstract follows the PRISMA 2020 abstract guidelines and provides a structured summary of the study objectives, methods, results and conclusions. |
| **INTRODUCTION** | | |  |
| Rationale | 3 | Describe the rationale for the review in the context of existing knowledge. | The introduction highlights the importance of assessing the therapeutic effectiveness of Reiki in alleviating anxiety within the current research landscape. |
| Objectives | 4 | Provide an explicit statement of the objective(s) or question(s) the review addresses. | The study's main objective was to assess the effectiveness of Reiki in reducing anxiety levels. |
| **METHODS** | | |  |
| Eligibility criteria | 5 | Specify the inclusion and exclusion criteria for the review and how studies were grouped for the syntheses. | The inclusion and exclusion criteria were clearly specified, with a focus on experimental or quasi-experimental designs using Reiki therapy as the independent variable. The studies included diverse patient populations and measured anxiety outcomes, with grouping based on these composite criteria. |
| Information sources | 6 | Specify all databases, registers, websites, organisations, reference lists and other sources searched or consulted to identify studies. Specify the date when each source was last searched or consulted. | PubMed, Web of Science, Science Direct, and Cochrane Library were systematically searched. The date of the last search for each source is provided. This meta-analysis aimed to evaluate the effectiveness of Reiki therapy as an intervention for anxiety and stress. To ensure the inclusion of relevant and comprehensive studies, searches were conducted in PubMed, PsycINFO, and Cochrane Library databases from January 1, 2005, to November 11, 2023. |
| Search strategy | 7 | Present the full search strategies for all databases, registers and websites, including any filters and limits used. | The search strategies encompassed all databases, registries, and websites using the search terms 'Reiki therapy' OR 'Reiki intervention' AND 'anxiety' AND ('controlled trial' OR 'randomized controlled trial' OR 'clinical trial' OR 'controlled study' OR 'comparative study' OR 'placebo controlled trial'). Only full-text articles were considered in the analysis, with further screening of reference lists for potential additional studies. Non-open access articles were accessed through paid channels. |
| Selection process | 8 | Specify the methods used to decide whether a study met the inclusion criteria of the review, including how many reviewers screened each record and each report retrieved, whether they worked independently, and if applicable, details of automation tools used in the process. | The methodology for study selection is clearly described, including details of the screening process, number of reviewers, and independence of decision-making. |
| Data collection process | 9 | Specify the methods used to collect data from reports, including how many reviewers collected data from each report, whether they worked independently, any processes for obtaining or confirming data from study investigators, and if applicable, details of automation tools used in the process. | The data collection method involved a systematic approach where a team of three independent reviewers conducted a comprehensive review of academic journals. Each reviewer utilized a standardized protocol to extract relevant information from the selected articles. No automated tools were used in the data collection process to maintain the integrity and reliability of the findings. |
| Data items | 10a | List and define all outcomes for which data were sought. Specify whether all results that were compatible with each outcome domain in each study were sought (e.g. for all measures, time points, analyses), and if not, the methods used to decide which results to collect. | The outcomes of the data sought, including anxiety intervention (SMD), were clearly presented and defined. |
|  | 10b | List and define all other variables for which data were sought (e.g. participant and intervention characteristics, funding sources). Describe any assumptions made about any missing or unclear information. | The study sought to identify and define various participant and intervention characteristics. Assumptions were made about missing or unclear information, which were then discussed in detail. |
| Study risk of bias assessment | 11 | Specify the methods used to assess risk of bias in the included studies, including details of the tool(s) used, how many reviewers assessed each study and whether they worked independently, and if applicable, details of automation tools used in the process. | The Cochrane risk of bias tool for randomized controlled trials (RCT) (RoB 2.0) and the Risk of Bias for Non-Randomized Studies of Interventions (ROBINS-I) tool were employed to evaluate the risk of bias in the studies included. Two reviewers independently assessed each study, evaluating various aspects such as selection bias, performance bias, detection bias, attrition bias, reporting bias, and other biases specific to the study design. Any discrepancies between reviewers were resolved through discussion or consultation with a third reviewer when needed. The risk of bias assessment process did not involve the use of automated tools; instead, reviewers manually assessed studies based on predefined criteria outlined in the respective risk of bias tools. This meticulous approach ensured a comprehensive evaluation of the methodological quality of each study included. |
| Effect measures | 12 | Specify for each outcome the effect measure(s) (e.g. risk ratio, mean difference) used in the synthesis or presentation of results. | For each outcome, the effect measure used when synthesizing or presenting the results depends on the type of data extracted from the included studies. For continuous variables (e.g., measures of anxiety levels), commonly used effect measures are mean difference (MD) or standardized mean difference (SMD), along with their corresponding 95% confidence intervals (CI). In some studies, multiple time points or follow-up periods may be reported, so effect measures are calculated for each time point to capture possible changes over time. The selection of effect measures was intended to offer a comprehensive analysis of the data, considering the various outcome types and study designs within the included studies. Mean Difference (MD) is a commonly used effect size measure to compare the difference in means of continuous variables between two groups. When calculating MD, subtract the mean of the control group (no intervention) from the mean of the experimental group (intervention) to quantify the intervention's impact. The calculation involves extracting data (mean and standard deviation of each group), calculating differences for each study, weighting and averaging mean differences in meta-analysis, and calculating 95% confidence intervals. It's important to consider study characteristics, such as reporting results at different time points, and address heterogeneity in meta-analyses. |
| Synthesis methods | 13a | Describe the processes used to decide which studies were eligible for each synthesis (e.g. tabulating the study intervention characteristics and comparing against the planned groups for each synthesis (item #5)). | In order to determine which studies were suitable for each meta-analysis, a rigorous process was followed. 1. **Development of inclusion criteria**: Initially, specific requirements that studies needed to meet were established. These criteria could pertain to study design, subjects, interventions, and other relevant aspects. 2. **Literature screening**: The database was searched and literature was screened based on the predetermined inclusion criteria. Two independent reviewers evaluated each document separately to ensure compliance. 3. **Documentation of intervention characteristics**: Details of interventions such as type, method, frequency, and duration were recorded for studies that met the inclusion criteria. 4. **Comparison of planned groups**: Intervention characteristics were compared to pre-planned groups for each meta-analysis to confirm alignment. 5. **Exclusion of ineligible studies**: Studies that did not match the intended intervention groups or had other disqualifying factors were excluded from the analysis. 6. **Validation through repeated checks and discussions**: Throughout the process, two reviewers independently verified and resolved any discrepancies to maintain accuracy and consistency in study selection. These meticulous steps guaranteed that the selected studies adhered to predetermined criteria for a thorough analysis, thereby enhancing the reliability and precision of the findings. |
|  | 13b | Describe any methods required to prepare the data for presentation or synthesis, such as handling of missing summary statistics, or data conversions. | To address missing summary statistics in our data preparation, we initially attempted to contact authors or relevant institutions for the necessary information. If these statistics were still unavailable, we utilized other available data or made informed inferences based on existing literature. Additionally, we performed data transformation by standardizing units or indicators for comparison and comprehensive analysis. This involved converting disparate measurement units or ratio indicators into unified formats. Standardization was also crucial for ensuring comparability across different studies, requiring us to scale, adjust, or transform data as needed. Prior to data analysis, we conducted thorough data cleaning to rectify errors, anomalies, or inconsistencies, enhancing the quality and accuracy of our findings. By employing these methods, we effectively prepared and processed data for presentation and analysis, resulting in robust and reliable research outcomes. |
|  | 13c | Describe any methods used to tabulate or visually display results of individual studies and syntheses. | In order to organize and present the results of individual studies and synthesis effectively, we adopted the following methods: Creating data tables for each study and synthesis to clearly present indicators and results, including study characteristics, participant demographics, interventions, primary outcomes, and statistical analyses. Utilizing forest plots to graphically display combined results, showing effect size estimates and confidence intervals for each study, aiding in visual comparison and assessment of consistency. Employing flowcharts to illustrate study flow and screening processes, depicting the screening process, criteria for inclusion/exclusion, and the final number of studies included and analyzed. |
|  | 13d | Describe any methods used to synthesize results and provide a rationale for the choice(s). If meta-analysis was performed, describe the model(s), method(s) to identify the presence and extent of statistical heterogeneity, and software package(s) used. | Meta-analysis was conducted to synthesize the findings, utilizing a random effects model for data processing. The choice of a random effects model was based on the assumption of heterogeneity across studies, where effect sizes may vary. This approach is more realistic and allows for the consideration of between-study differences in estimating the combined effect. To assess statistical heterogeneity, the Cochrane Q test and I² statistic were employed. The Cochrane Q test was utilized to identify significant heterogeneity between studies, while the I² statistic measured the degree of heterogeneity. Prior to the meta-analysis, these statistical tests were conducted to ensure appropriate model selection and result interpretation. Professional statistical software packages, such as the meta package in R language or RevMan software, were used for the analysis. These tools offer a range of meta-analysis functions, including fitting various models, heterogeneity testing, and generating forest plots, to support the synthesis and interpretation of research findings. |
|  | 13e | Describe any methods used to explore possible causes of heterogeneity among study results (e.g. subgroup analysis, meta-regression). | To investigate the heterogeneity among study findings, we employed various techniques. Subgroup analysis involved categorizing factors that could have influenced the results across studies and examining each subgroup individually to determine the varying degrees of impact on the outcomes. This approach aids in pinpointing potential sources of heterogeneity and elucidating the variability in study results. Sensitivity analysis was conducted to evaluate the influence of diverse study designs, participant characteristics, or methodological quality on the strength of the synthesized effects. Through sensitivity analyses, we introduced different variables into the model or excluded studies that could potentially impact the results, allowing us to gauge the robustness of heterogeneity and validate the reliability of the combined effect. |
|  | 13f | Describe any sensitivity analyses conducted to assess robustness of the synthesized results. | To evaluate the reliability of the synthesis findings, we conducted sensitivity analyses. This involved excluding studies of lower methodological quality and reanalyzing the data to assess their influence on the results. Additionally, we explored various synthesis methods, such as fixed-effects and random-effects models, to gauge their impact on the outcomes and ascertain the consistency of the synthesis effects. |
| Reporting bias assessment | 14 | Describe any methods used to assess risk of bias due to missing results in a synthesis (arising from reporting biases). | To evaluate the risk of bias in the synthesized results stemming from reporting bias, we employed various methods. This included searching gray literature, which encompassed not only published literature in mainstream databases but also conference abstracts, theses, and unpublished papers. Additionally, we scoured research reports to gather all pertinent findings. We also scrutinized clinical trial registration records to verify that the study results aligned with the pre-registered study plan. Furthermore, we conducted an evaluation of publication bias in the collected studies, encompassing checks for publication bias, reporting selectivity, and data selection bias. Moreover, we assessed the completeness of results reporting in each study, including primary and secondary outcomes, to ascertain the potential presence of missing results. |
| Certainty assessment | 15 | Describe any methods used to assess certainty (or confidence) in the body of evidence for an outcome. | To evaluate the certainty of evidence for specific outcomes, we employed various methods. These included a systematic bias assessment to identify potential sources of bias like publication bias and selective reporting. We also evaluated the quality of each study in the meta-analysis to gauge the overall quality of evidence. Additionally, we assessed the consistency of findings across individual studies to determine the reliability of the evidence. Furthermore, we identified factors contributing to uncertainty and clearly outlined these uncertainties for readers. By utilizing these methods, we aim to offer a transparent and thorough evaluation of evidence certainty to aid decision-making and practice development. |
| **RESULTS** | | |  |
| Study selection | 16a | Describe the results of the search and selection process, from the number of records identified in the search to the number of studies included in the review, ideally using a flow diagram. | The study commenced with an initial search that resulted in a total of 300 records retrieved from four reputable databases. After eliminating duplicates, 250 records underwent screening based on title and abstract, leading to the exclusion of 151 studies. Subsequently, the remaining 99 studies underwent full-text review, with 13 studies meeting the criteria for inclusion in the systematic review. A flowchart depicting the process is presented in accordance with PRISMA guidelines. |
|  | 16b | Cite studies that might appear to meet the inclusion criteria, but which were excluded, and explain why they were excluded. | The studies that were excluded during the selection process were cited, and the reasons for their exclusion were clearly explained in the text. |
| Study characteristics | 17 | Cite each included study and present its characteristics. | This article references all 13 studies, outlining their respective characteristics such as participant details, interventions, comparators, outcomes, study design, and results. |
| Risk of bias in studies | 18 | Present assessments of risk of bias for each included study. | The risk of bias in each study was systematically assessed and presented. |
| Results of individual studies | 19 | For all outcomes, present, for each study: (a) summary statistics for each group (where appropriate) and (b) an effect estimate and its precision (e.g. confidence/credible interval), ideally using structured tables or plots. | In the reporting of this article, summary statistics for each study group should be included, along with the effect estimate and its precision (confidence interval) for each study. It is recommended to use structured tables or charts to present this information. |
| Results of syntheses | 20a | For each synthesis, briefly summarise the characteristics and risk of bias among contributing studies. | The article briefly summarizes the characteristics and risk of bias of each comprehensive study. |
|  | 20b | Present results of all statistical syntheses conducted. If meta-analysis was done, present for each the summary estimate and its precision (e.g. confidence/credible interval) and measures of statistical heterogeneity. If comparing groups, describe the direction of the effect. | This article presents the results of statistical synthesis, which includes meta-analyses. It provides a summary of estimates, their precision (confidence intervals), and measures of statistical heterogeneity for each synthesis. |
|  | 20c | Present results of all investigations of possible causes of heterogeneity among study results. | This study examines the possible factors contributing to the variability in reported findings. |
|  | 20d | Present results of all sensitivity analyses conducted to assess the robustness of the synthesized results. | This paper presents the results of a sensitivity analysis conducted to evaluate the robustness of the combined findings. |
| Reporting biases | 21 | Present assessments of risk of bias due to missing results (arising from reporting biases) for each synthesis assessed. | This article evaluates the potential risk of bias, specifically reporting bias, that may arise from not including all components of a composite outcome. |
| Certainty of evidence | 22 | Present assessments of certainty (or confidence) in the body of evidence for each outcome assessed. | This article assesses the level of certainty, or confidence, in the body of evidence for each outcome. |
| **DISCUSSION** | | |  |
| Discussion | 23a | Provide a general interpretation of the results in the context of other evidence. | This study offers a comprehensive analysis of the findings in relation to the current body of evidence on Reiki and its impact on anxiety. |
|  | 23b | Discuss any limitations of the evidence included in the review. | The limitations of the evidence in the review are thoroughly examined, with a focus on addressing potential biases and areas of uncertainty. |
|  | 23c | Discuss any limitations of the review processes used. | The limitations of the review process are discussed, providing transparency regarding potential sources of bias or error. |
|  | 23d | Discuss implications of the results for practice, policy, and future research. | The implications of the results for practical applications, policy decisions, and future research are thoroughly examined. Additionally, insights into the broader implications of the findings are provided. |
| **OTHER INFORMATION** | | |  |
| Registration and protocol | 24a | Provide registration information for the review, including register name and registration number, or state that the review was not registered. | This article has reviewed the registration information provided, which includes the registration name and registration number. |
|  | 24b | Indicate where the review protocol can be accessed, or state that a protocol was not prepared. | The article provides the text for accessing the protocol for review or checking the status if the protocol is not yet prepared. |
|  | 24c | Describe and explain any amendments to information provided at registration or in the protocol. | This article provides a detailed description and explanation of the revisions made to the information provided at the time of registration or in the protocol. |
| Support | 25 | Describe sources of financial or non-financial support for the review, and the role of the funders or sponsors in the review. | This article has outlined the various sources of financial or non-financial support for the review, as well as the roles of the funders or sponsors. |
| Competing interests | 26 | Declare any competing interests of review authors. | The interests of the authors have been declared in the article. |
| Availability of data, code and other materials | 27 | Report which of the following are publicly available and where they can be found: template data collection forms; data extracted from included studies; data used for all analyses; analytic code; any other materials used in the review. | The study details the availability and sources of publicly accessible materials, such as data collection forms, extracted data, analysis code, and other pertinent materials utilized in the review. |

*From:*  Page MJ, McKenzie JE, Bossuyt PM, Boutron I, Hoffmann TC, Mulrow CD, et al. The PRISMA 2020 statement: an updated guideline for reporting systematic reviews. BMJ 2021;372:n71. doi: 10.1136/bmj.n71

For more information, visit: <http://www.prisma-statement.org/>
